# Angiotensin-Converting Enzyme 2 Potentiates SARS-CoV-2 Infection by Antagonizing Type I Interferon Induction and Its Down-Stream Signaling Pathway

**DOI:** 10.1128/msphere.00211-22

**Published:** 2022-07-12

**Authors:** Jian Chen, Jian Liu, Zhilu Chen, Haoran Peng, Cuisong Zhu, Daobin Feng, Shuye Zhang, Ping Zhao, Xiaoyan Zhang, Jianqing Xu

**Affiliations:** a Clinical Center for Bio-Therapy, Zhongshan Hospital, Fudan University (Xiamen Branch), Shanghai, China; b Department of Microbiology, Second Military Medical University, Shanghai, China; c Shanghai Public Health Clinical Center & Institutes of Biomedical Sciences, Shanghai Medical College, Fudan University, Shanghai, China; University of Maryland School of Medicine

**Keywords:** ACE2, SARS-CoV-2, IRF3, STAT2, Interferon signaling

## Abstract

The innate interferon (IFN) response constitutes the first line of host defense against viral infections. It has been shown that IFN-I/III treatment could effectively contain severe acute respiratory syndrome coronavirus 2 (SARS-CoV-2) replication *in vitro*. However, how SARS-CoV-2 survives through the innate antiviral mechanism remains to be explored. Our study uncovered that human angiotensin-converting enzyme 2 (ACE2), identified as a primary receptor for SARS-CoV-2 entry, can disturb the IFN-I signaling pathway during SARS-CoV-2 infection in human lung cells. We identified that ACE2 was significantly upregulated by SARS-CoV-2 and Sendai virus (SeV) infection, and exogenous expression of ACE2 suppressed IFN-I production in a dose-dependent manner. Mechanistically, ACE2 disrupted poly (I:C)-mediated inhibition of SARS-CoV2 replication by antagonizing IFN-I production by blocking IRF3 phosphorylation and nuclear translocation. Moreover, ACE2 quenched the IFN-mediated antiviral immune response by degrading endogenous STAT2 protein, inhibiting STAT2 phosphorylation and nuclear translocation. Interestingly, IFN-inducible short ACE2 (dACE2 or MIRb-ACE2) can also be induced by virus infection and inhibits the IFN signaling. Thus, our findings provide mechanistic insight into the distinctive role of ACE2 in promoting SARS-CoV-2 infection and enlighten us that the development of interventional strategies might be further optimized to interrupt ACE2-mediated suppression of IFN-I and its signaling pathway.

**IMPORTANCE** Efficient antiviral immune responses against SARS-CoV-2 infection play a key role in controlling the coronavirus diseases 2019 (COVID-19) caused by this virus. Although SARS-CoV-2 has developed strategies to counteract the IFN-I signaling through the virus-derived proteins, our knowledge of how SARS-CoV-2 survives through the innate antiviral mechanism remains poor. We herein discovered the distinctive role of ACE2 as a restraining factor of the IFN-I signaling in facilitating SARS-CoV-2 infection in human lung cells. Both full-length ACE2 and truncated dACE2 can antagonize IFN-mediated antiviral response. These findings are key to understanding the counteraction between SARS-CoV-2 pathogenicity and the host antiviral defenses.

## INTRODUCTION

The ongoing pandemic of coronavirus diseases 2019 (COVID-19) caused by the severe acute respiratory syndrome coronavirus 2 (SARS-CoV-2) has caused more than 452 million human infections, with more than 6.0 million deaths worldwide (https://covid19.who.int/). However, how SARS-CoV-2 enters host target cells and survives through the innate antiviral mechanism remains to be explored. The innate interferon (IFN) response constitutes the first line of host defense against viral infections. It has been shown that IFN-I/III treatment could effectively contain SARS-CoV-2 replication *in vitro* (1) and *in vivo* (2). Therefore, it is rationalized that SARS-CoV-2 has developed strategies to counteract the IFN-I signaling. Compared with SARS-CoV and Middle East respiratory coronavirus (MERS-CoV), SARS-CoV-2 nsp1 and nsp6 proteins suppressed IFN-I signaling more efficiently (3). The SARS-CoV-2 nsp12 protein attenuates IFN-I production by inhibiting IRF3 nuclear translocation (4), nsp14 abolishes the induction of interferon-stimulated genes (ISGs) via a translational shutdown (5), nucleocapsid protein represses retinoic acid-inducible gene I (RIG-I)-mediated IFN-β production (6), and membrane glycoprotein M antagonizes the mitochondrial antiviral signaling protein (MAVS)-mediated innate antiviral response (7). SARS-CoV-2 is an enveloped, positive-sense, single-stranded RNA beta-coronavirus. The entry of SARS-CoV-2 into its target cells, such as lung alveolar or bronchial cells, is reported to depend on its receptor angiotensin-converting enzyme 2 (ACE2) (8, 9). However, a recent report has demonstrated that SARS-CoV-2 could efficiently enter a human alveolar basal epithelial carcinoma cell line, A549 cells, which expressed a low level of ACE2, but its replication was abolished (10). In contrast, the exogenous constitutive expression of human ACE2 in wild type (WT) A549 cells confers SARS-CoV-2 to successful replication upon entering these cells (11). Interestingly, we observed that ACE2 expression was upregulated by SARS-CoV-2 (Fig. S1A) and Sendai virus (SeV) (Fig. S1B) infection in WT A549 cells, which is following the observation that the ACE2 expression was evidenced during SARS-CoV-2 infection of the respiratory epithelia in COVID-19 (12) (Fig. S1C, adapted from Nawijn and Timens [13]). From this perspective, we speculate that there may exist an ACE2-mediated mechanism antagonizing host antiviral signaling to facilitate SARS-CoV-2 replication.

**FIG 1:**
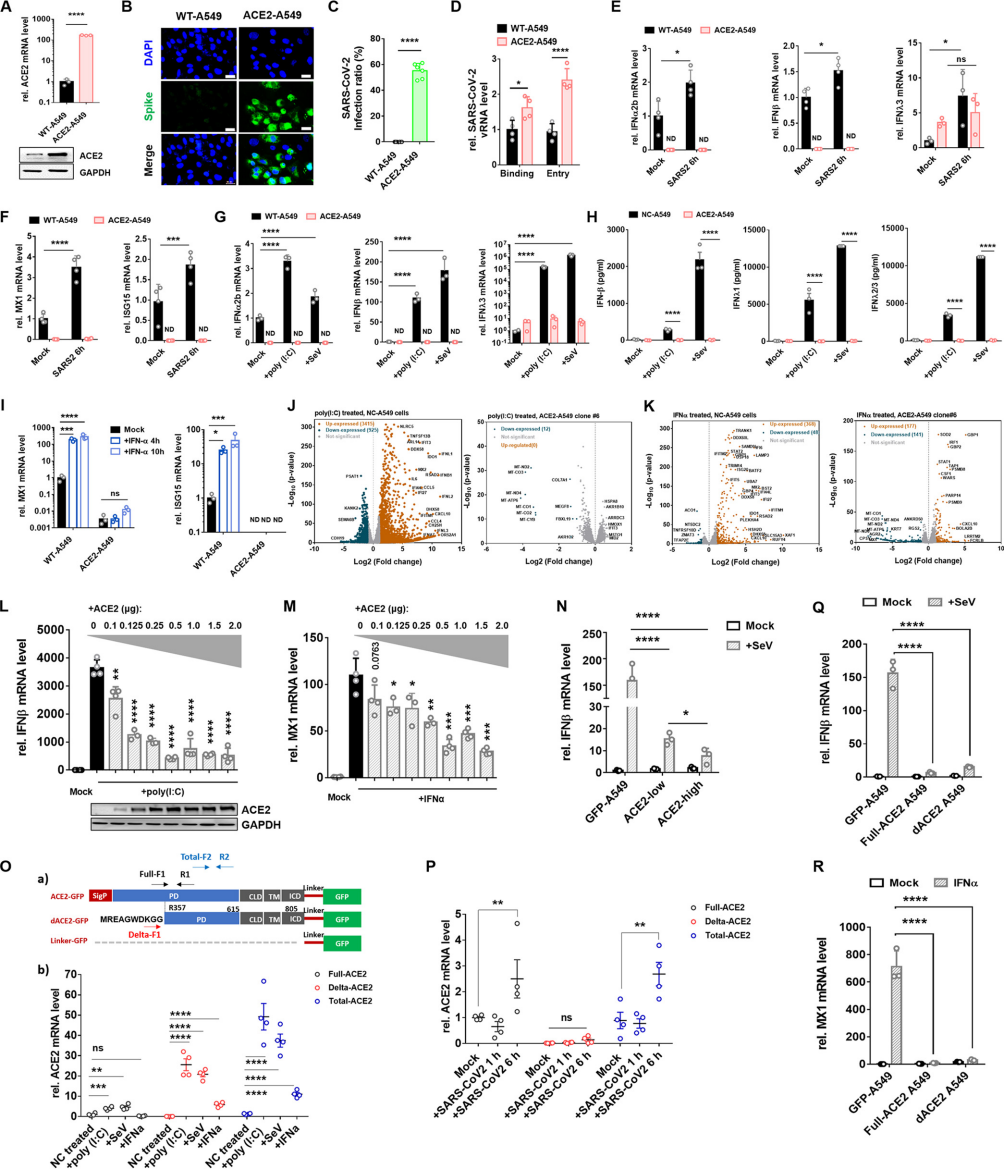
ACE2 disrupted the IFN-I induction and response signaling pathway. (A) RT-qPCR and Western blot analyses for ACE2 expression in the WT and ACE2-A549 cells. The stable A549 cell line expressing the full-length of human ACE2 was constructed using a lentiviral vector. (B and C) Immunofluorescence image and statistical analyses for authentic SARS-CoV-2 infection in WT and ACE2-A549 cells. Cells were infected with SARS-CoV-2 for 48 h. MOI = 2; Blue, DAPI; Green, Spike. Scale bars, 20 μm; ****, *P < *0.0001. (D) RT-qPCR analysis for SARS-CoV-2 vRNA level in WT and ACE2-A549 cells. For viral attachment assay, cells were incubated with authentic SARS-CoV-2 on ice for 1 h; for viral entry assay, cells were incubated with authentic SARS-CoV-2 on ice for 1 h, followed by additional 1 h of incubation in 37°C. MOI = 10. All cells were washed with cold 1 × PBS three times after the virus treatment. The level of viral RNA was detected after the lysis cells were extracted. (E) RT-qPCR analysis for the expression of IFN-α2b, IFN-β, and IFN-λ3 in WT and ACE2-A549 cells. Cells were infected with SARS-CoV-2 for 6 h. MOI = 2. ND, not detected; ns, no significance; *, *P < *0.05. (F) The expression of MX1 and ISG15 in WT and ACE2-A549 cells infected with or without SARS-CoV-2 for 6 h. MOI = 2. ND, not detected; ***, *P < *0.001; ****, *P < *0.0001. (G) The expression of IFN-α2b, IFN-β, and IFN-λ3 in WT and ACE2-A549 cells treated with control, 5 μg/mL poly (I:C), or SeV (MOI = 1) for 10 h. ND, not detected; ****, *P < *0.0001. (H) Interferons production in ACE2-A549 cells was compared with linker-GFP negative control (NC) A549 cells. Production of IFNβ, IFNλ1, and IFNλ2/3 in culture supernatants was quantified by Cytometric Bead Array (CBA) assay after incubation with 5 μg/mL poly (I:C) or SeV (MOI = 1) for 10 h. (I) RT-qPCR analysis for the expression of MX1 and ISG15 in WT and ACE2-A549 cells. Cells were treated with 1000 U/mL IFN-α for 4 h and 10 h. ND, not detected; ns, no significance; *, *P < *0.05; ***, *P < *0.001; ****, *P < *0.0001. (J and K) RNA-Seq analysis of mock and poly (I:C)-stimulated NC-A549 cells and ACE2-A549 clone#6 cells at 10 h post-stimulation (J); RNA-Seq analysis of mock and IFNα-stimulated NC-A549 cells and ACE2-A549 clone#6 cells at 10 h post-stimulation (K). Three replicates in each group. Volcano plot of genes differentially expressed in mock and stimulated cells. Significant DEGs were identified with a *P*-value ≤ 0.05 and a log_2_ (fold change) ≥1. (L and M) RT-qPCR analysis for the expression of IFNβ and MX1 in U-251 MG cells. U-251 MG cells were transfected with increasing amounts of vectors expressing ACE2 or a control vector for 24 h. The cells were then treated with 5 μg/mL poly (I:C) (L) or 1000 U/mL IFNα (M) for an additional 10 h and assayed for mRNA level. The indicated protein expression levels were analyzed by Western blotting. *, *P < *0.05; **, *P < *0.01; ***, *P < *0.001; ****, *P < *0.0001. (N) RT-qPCR analysis for the expression of IFNβ in NC-A549, ACE2-low, and high A549 cells at 10 h post-SeV infection (MOI = 1). *, *P < *0.05; ****, *P < *0.0001. (O, panel a) Schematics of predicted long and short ACE2 protein isoforms, linker-GFP control, primer design, and vector constructs. ACE2 is a single-span transmembrane protein with a signal peptide (SigP) of 17 aa and four functional domains-peptidase domain (PD, aa 18–615), collectrin-like domain (CLD, aa 616–740), transmembrane domain (TM, aa 741–761) and intracellular domain (ICD, aa 762–805). In dACE2, the signal peptide is not predicted; the peptidase domain starts from aa R357; the first 356 aa are replaced by 10 aa of a unique protein sequence (MREAGWDKGG). (O, panel b) Medians and individual data points showing relative expression of full-ACE2 (hollow black circles), delta-ACE2 (red hollow circles), and total-ACE2 (hollow blue circles) transcripts in WT A549 cells stimulated with 5 μg/mL poly (I:C), SeV (MOI = 1), or 1000 U/mL IFNα for 10 h. *n* = 4 independent experiments. ns, no significance; **, *P < *0.01; ***, *P < *0.001; ****, *P < *0.0001. (P) Medians and individual data points showing relative expression of full-ACE2 (hollow black circles), delta-ACE2 (red hollow circles), and short ACE2 (hollow blue circles) transcripts in WT A549 cells infected with SARS-CoV-2 for 1 and 6 h. *n* = 4 independent experiments. ns, no significance; **, *P < *0.01. (Q and R) RT-qPCR analysis for the expression of IFNβ (Q) and MX1 (R) in NC-A549, full-ACE2-A549, and delta-ACE2-A549 cells. The cells were infected with SeV (MOI = 1) (Q) or treated with 1000 U/mL IFNα (R) for 10 h. ****, *P < *0.0001.

10.1128/msphere.00211-22.1 FIG S1 ACE2 antagonizes IFN-I signaling. (A and B) RT-qPCR analysis for the expression of ACE2 in WT A549 cells. Cells were infected with SARS-CoV-2 (MOI = 2) for 1 h and 6 h (A) or infected with SeV (MOI = 1) (B) for 10 h. *, *P < *0.05; **, *P < *0.01; ****, *P < *0.0001. (C) Putative events during SARS-CoV-2 infection of the respiratory tract (this proposed model was adapted from Nawijn and Timens[13]). The antiviral responses induced upon SARS-CoV-2 infection induce a marked upregulation of ACE2 expression, allowing the SARS-CoV-2 to spread across the respiratory mucosa and into the parenchyma of the lung, where it can infect type-2 alveolar epithelial cells. (D) Schematic illustration of stable cell line construction. GFP^+^ cells were sorted by fluorescence-activated cell sorting (FACS). (E) RT-qPCR analysis for SARS-CoV-2 vRNA level in WT and ACE2-H1299 cells. For viral attachment assay, cells were incubated with authentic SARS-CoV-2 on ice for 1 h; for viral entry assay, cells were incubated with authentic SARS-CoV-2 on ice for 1 h, followed by additional 1 h incubation in 37°C. MOI = 10. All cells were washed with cold 1 × PBS three times after the virus treatment. The level of viral RNA was detected after the lysis cells were extracted. (F) Representative RT-qPCR amplification curves supplemented to Fig. 1D. (G) The expression of IL-1β, IL-6, and TNF-α in WT and ACE2-A549 cells infected with or without SARS-CoV-2 for 6 h. MOI = 2. *, *P < *0.05; **, *P < *0.01. (H) RT-qPCR analysis for the expression of MX1 and ISG15 in WT and ACE2-A549 cells treated with control, 5 μg/mL poly (I:C), or SeV (MOI = 1) for 10 h. ND, not detected; ***, *P < *0.001; ****, *P < *0.0001. (I) RT-qPCR analysis for the expression of IL-1β and IL-6 in WT and ACE2-A549 cells treated with control or 5 μg/mL poly (I:C) for 4 h. *, *P < *0.05; ****, *P < *0.0001. (J) RT-qPCR analysis for the expression of IFN-α2b, IFN-β, and IFN-λ3 in WT and ACE2-KO A549 cells treated with control or 5 μg/mL poly (I:C) for 10 h. ns, no significance; ***, *P < *0.001; ****, *P < *0.0001. (K) RT-qPCR analysis for the expression of MX1 and ISG15 in WT and ACE2-KO A549 cells treated with 1000 U/mL IFN-α for 10 h. ND, not detected; ns, no significance; ***, *P < *0.001; ****, *P < *0.0001. (L) RT-qPCR analysis for the expression of IFN-α2b, IFN-β, and IFN-λ3 in NC-A549 and ACE2-A549 clonal cell lines treated with mock or 5 μg/mL poly (I:C) and SeV (MOI = 1) for 10 h. ****, *P < *0.0001. (M) RT-qPCR analysis for the expression of MX1 and ISG15 in NC-A549 and ACE2-A549 clonal cell lines treated with mock or 1000 U/mL IFNα for 10 h. ns, no significance; ****, *P < *0.0001. (N and O) RT-qPCR analysis for the expression of IFN-β and IFN-λ3 in NC- and ACE2-expressing U-251 MG (N) or H1299 cells (O) treated with mock or 5 μg/mL poly (I:C) for 10 h. ****, *P < *0.0001. Download FIG S1, TIF file, 2.6 MB. Copyright © 2022 Chen et al. 2022 Chen et al. https://creativecommons.org/licenses/by/4.0/ This content is distributed under the terms of the Creative Commons Attribution 4.0 International license.

## RESULTS

To address the role of ACE2 in regulating host antiviral signaling, we first generated a stable cell line of A549 expressing the full-length of human ACE2 using a lentiviral vector designated ACE2-A549 (Fig. S1D). RT-qPCR and Western immunoblotting analyses revealed a significant elevation of ACE2 mRNA and ACE2 protein levels in ACE2-A549 cells compared to WT A549 cells (Fig. 1A). Consistent with previous studies, WT A549 cells were not susceptible to SARS-CoV-2. However, viral replication was significantly enhanced in ACE2-A549 cells at 48 h after authentic SARS-CoV-2 infection (Fig. 1B and C). We next examined the viral attachment and entry efficacy of SARS-CoV-2 in WT and ACE2-A549. SARS-CoV-2 binding and endocytosis were observed in WT A549 cells, with a moderate increase in ACE2-expressing cells (Fig. 1D). A similar observation was also obtained in NCI-H1299 cells (Fig. S1E). The representative amplification curves of RT-qPCR were shown in Fig. S1F, revealing a quite minor difference in Ct value between WT and ACE2-expressing cells. These data indicate that the robust SARS-CoV-2 replication in ACE2-A549 cells is unlikely to be merely determined by ACE2-mediated endocytosis enhancement.

It has been demonstrated that a host factor, such as receptor tyrosine kinase, AXL, promotes RNA virus replication by antagonizing type I interferon signaling (14, 15). Thus, we deciphered whether ACE2 can regulate the IFN-I signaling pathway. We first examined the innate cytokine responses during the early stage of SARS-CoV-2 infection in A549 cells. IFN-α2b, IFN-β, and IFN-λ3 were significantly upregulated in WT A549 cells at 6 h after SARS-CoV-2 infection (Fig. 1E). Conversely, IFN-α2b and IFN-β were marginally detected in mock-infected or SARS-CoV-2 infected ACE2-A549 cells. In addition, we hardly observed a significant increase of IFN-λ3 mRNA in response to SARS-CoV-2 infection compared with that in mock-infected ACE2-A549 cells (Fig. 1E). These observations suggest that ACE2 may be able to significantly regulate endogenous type I/III IFN responses during SARS-CoV-2 infection. A significant upregulation of interferon-induced gene expressions (ISGs) expression, such as MX1 and ISG15, was consistently identified in WT A549 cells but not in ACE2-A549 cells at 6 h after SARS-CoV-2 infection (Fig. 1F). Interestingly, following a previous study (16), we detected the elevated mRNA induction of interleukin (IL)-1β, IL-6, and tumor necrosis factor (TNF) in response to SARS-CoV-2 infection in ACE2-A549 cells compared to WT A549 cells (Fig. S1G). In addition, we further tested those cells after being transfected or infected with poly (I:C) or SeV, respectively, both of which are often used to stimulate antiviral signaling pathways. Upon poly (I:C) transfection and SeV infection, the induction of IFN-α2b, IFN-β, and IFN-λ3 mRNA (Fig. 1G) and protein secretion (Fig. 1H) was dramatically impaired in ACE2-A549 cells, as well as the production of ISGs, including MX1 and ISG15 expression (Fig. S1H). Of note, IL-1β and IL-6 were markedly induced in ACE2-A549 cells compared to WT A549 cells at the baseline and 10 h after poly (I:C) treatment (Fig. S1I). Genetic ablation of ACE2 enhanced the antiviral innate immune response without or with poly (I:C) treatment (Fig. S1J and K). To investigate whether ACE2 also regulated the IFN-I response signaling, WT or ACE2-A549 cells were treated with recombinant human IFN-α for 4 and 10 h. We found that MX1 and ISG15 mRNA expression was significantly suppressed in ACE2-A549 cells compared to WT A549 cells (Fig. 1I). Thus, ACE2 may play a regulatory role in both the production and downstream responses of the IFN-I signaling pathway. To exclude the possible bias from a mixed population of cells and one cell line, we thereby established low passage single cell-derived clones (clone#1, clone#5, and clone#6) and treated these three clonal cell lines with poly(I:C), SeV, or IFNα. RNA-Seq (clone#6) and RT-qPCR (clone#1, clone#5, and clone#6) analyses revealed that IFN induction (Fig. 1J and Fig. S1L) and responses (Fig. 1K and S1M) were significantly decreased in ACE2-expressing clonal cells, pLV-Linker-GFP cells as the negative control (NC) cells. Next, we assessed the role of ACE2 in regulating IFN signaling in two additional cell lines, NCI-H1299 (Human non-small cell lung cancer cell line) and U-251 MG (Human glioblastoma cell line). Consistent with the results in A549 cells, ACE2-expressing negatively regulated IFN signaling in both U-251 MG (Fig. S1N) and H1299 (Fig. S1O) cells. These results reveal an unconventional negative regulation mechanism by which ACE2 suppresses IFN signaling induced by double-strand RNA (dsRNA) or RNA virus infection and consequently abolishes host antiviral responses. To discern the physiological relevance of this finding to the development of COVID-19, we transfected U-251 MG cells with an increasing dose of ACE2 (0.1, 0.125, 0.25, 0.5, 1, and 2 μg) or a negative control vector for 24 h, then stimulated the cells with poly (I:C), or IFNα for an additional 10 h. We found that the inhibitory effects of ACE2 on IFN production and IFN responses were dose-dependent, and ACE2 already exerted a significant inhibitory effect on the IFN-I signaling at a concentration as low as 0.1 μg/mL (Fig. 1L and M). Furthermore, we sorted two groups of A549 cells with low or high ACE2 protein from ACE2-expressing A549 cells (fresh constructed cell line) using fluorescence-activated cell sorting (FACS). Cells with high ACE2 expression exhibited more apparent inhibitory effects on IFNβ production than cells with minor ACE2 (Fig. 1N). These data suggest a significant correlation between the elevated amounts of ACE2 protein and the inhibitory effects on IFN-I signaling in both U-251 MG and A549 cells. Overall, these data explain clinical observations on the dramatically skewed responses from IFN to inflammatory responses (12, 17, 18).

It has been shown that ACE2 is a human interferon-stimulated gene in the airway epithelial cells (19), which is reported to be a novel, transcriptionally independent truncated isoform of ACE2 (dACE2 or MIRb-ACE2) and could be induced by IFNs and viruses (20–22). We next tested if the IFN-induced truncated protein is also induced in our system and if it also shows inhibitory potential. Using custom-designed assays, we detected the expression of full-ACE2, dACE2, and total-ACE2 in A549 cells at baseline or after 10 h of stimulating with SeV, poly (I:C), and IFNα. Following previous studies (19–22), dACE2 was significantly induced by IFNα and can also be upregulated by poly (I:C) and SeV stimulation (Fig. 1O). However, full-ACE2 was induced by poly (I:C) and SeV, but not IFNα stimulation (Fig. 1O), suggesting that full-ACE2 can be induced by an RNA virus directly. Alternatively, one plausible explanation is that poly (I:C) and SeV stimulation have the potential to induce IFN production and thereby stimulate dACE2 production rapidly. This chime with the data obtained from SARS-COV-2 infection, in which full-ACE2 was induced at the early stage (6 h) of infection (Fig. 1P). Ng et al. (22) also reported that dACE2 was induced at a late stage (24 h) of SARS-COV-2 infection in Calu-3 cells. These results sparked our interest in evaluating the role of dACE2 in the regulation of IFN signaling. Thus, we further exogenous expressed dACE2 in A549 cells and established a dACE2-A549 cell line, pLV-linker-GFP-A549 cells, as a negative control (NC) cells. Compared to full-ACE2-A549 cells, expressing dACE2 can also inhibit IFNβ and MX1 production after SeV infection (Fig. 1Q) or IFNα stimulation (Fig. 1R). Altogether, these results confirm that both full-length and truncated dACE2 can regulate the IFN signaling during viral infection.

**FIG 2:**
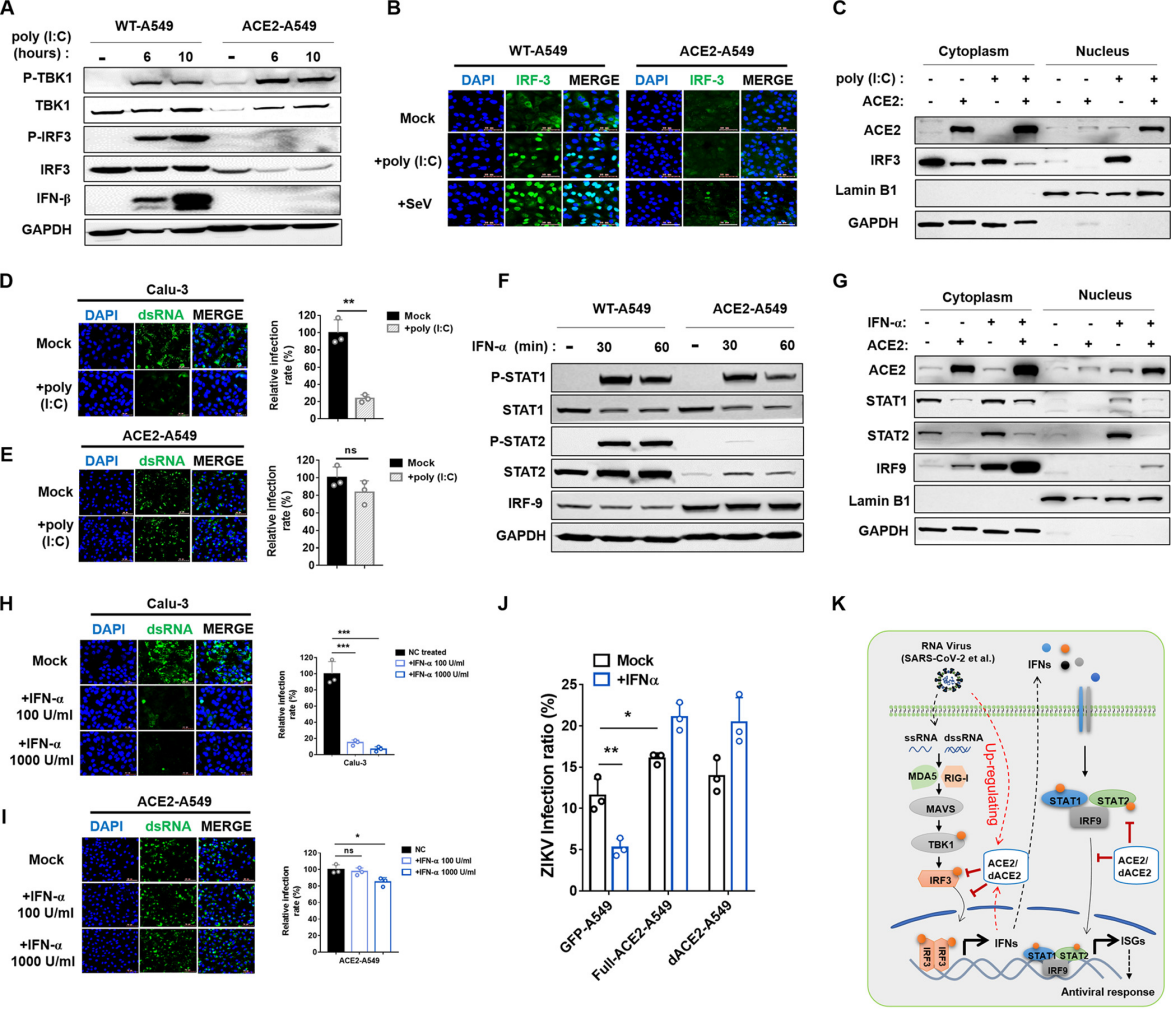
ACE2 inhibits IFN-mediated antiviral responses by suppressing the phosphorylation and nuclear translocation of IRF3 and STAT2. (A) Western blot analysis for endogenous level and phosphorylation of TBK1 and IRF3 in the WT and ACE2-A549 cells treated with control, or 5 μg/mL poly (I:C) for 6 h and 10 h. (B and C) Immunofluorescence (B) and Western blot (C) analyses for protein expression and nuclear translocation of IRF3. WT and ACE2-A549 cells were treated with control, 5 μg/mL poly (I:C), or SeV (MOI = 1) for 6 h and then immunostained with the indicated antibodies. Blue, DAPI; Green, IRF3; Scale bars, 50 μm. (D and E) Immunofluorescence image and statistical analyses for authentic SARS-CoV-2 infection in Calu-3 (D) and ACE2-A549 (E) cells. Cells were pretreated with or without 5 μg/mL poly (I:C) for 10 h, then were infected with SARS-CoV-2 for an additional 48 h. MOI = 2; Blue, DAPI; Green, dsRNA. Scale bars, 50 μm; ns, no significance; **, *P < *0.01. (F) Western blot analysis for endogenous level and phosphorylation of STAT1, STAT2, and IRF9 in the WT and ACE2-A549 cells treated with or without 1000 U/mL IFN-α for 30 min and 60 min. (G) Western blot analysis for nuclear translocation of ACE2, STAT1, STAT2, and IRF9. WT and ACE2-A549 cells were treated with control or 1000 U/mL IFN-α for 2 h, then immune-stained with the indicated antibodies. (H and I) Immunofluorescence image and statistical analysis for authentic SARS-CoV-2 infection in Calu-3 (H) and ACE2-A549 (I) cells. Cells were pretreated with or without IFN-α in the indicated dose for 10 h and then infected with SARS-CoV-2 for 48 h. MOI = 2; Blue, DAPI; Green, dsRNA. Scale bars, 50 μm. ns, no significance; *, *P < *0.05; ***, *P < *0.001; ****, *P < *0.0001. (J) Zika virus (ZIKV) infection in NC-A549, full-ACE2, and delta-ACE2 A549 cells. Cells were pretreated with or without 1000 U/mL IFN-α for 10 h and then were infected with ZIKV for an additional 24 h. MOI = 2; *, *P < *0.05; **, *P < *0.01. (K) Overview of the critical role ACE2 plays in regulating IFN-I signaling and promoting virus infection. This scheme illustrates that virus infection promotes ACE2 expression, inhibiting IFN-I induction and its downstream antiviral response signaling by targeting IRF3 and STAT2.

We then explored the underlying mechanism by which ACE2 is a host regulatory factor for type I IFN responses. The dsRNA, which is generated during coronavirus genome replication and transcription, could be recognized by RIG-I and/or melanoma differentiation gene 5 (MDA5) in the cytoplasm (23, 24). Then IFN-α/β was activated in the axis of the MAVS-TBK1/IKKi-IRF3/7 signaling pathway, which prompted us to test whether ACE2 modulates TBK1 and IRF3 phosphorylation, which are two key steps in the IFN induction pathway. We found that overexpression of ACE2 significantly downregulated endogenous and exogenous IRF3 protein and suppressed IRF3 phosphorylation (Fig. 2A and S2A) but did not affect IRF3 mRNA expression in ACE2-expressing A549 cells treated with poly (I:C) or SeV (Fig. S2B). NC-293T cells and ACE2-expressing 293T cells exhibited comparable IRF3 levels after IRF3 transfection (Fig. S2C). In contrast, endogenous TBK1, TBK1 phosphorylation, and the phosphorylation of JNK, P38, and ERK (Fig. S2D) were partially enhanced. Further immunofluorescence and Western immunoblotting analyses showed that in mock-treated A549 cells, IRF3 was distributed in the cytoplasm with or without ACE2 overexpressing (Fig. 2B, row 1). However, poly (I:C) and SeV-induced IRF3 nuclear translocation was severely impaired in cells overexpressing ACE2 (Fig. 2B, rows 2 and 3, and Fig. 2C). We then cotransfected IRF3-5D (a constitutively active IRF3 mutant) with IFN-β promoter and determined the activation of IFN-β promoter in NC-293T cells and ACE2-expressing 293T cells. Luciferase reporter assays showed that overexpression of ACE2 significantly inhibited IRF3-5D-triggered IFN-β promoter activation (Fig. S2E). These observations emphasized the critical role of ACE2 in targeting the downstream of the TBK1 signaling pathway and suppressing IFN production by reducing IRF3 protein and blocking IRF3 phosphorylation and nuclear translocation. Next, we investigated whether ACE2 could suppress poly (I:C)-mediated inhibition of SARS-CoV-2 replication. ACE2-A549 and Calu-3 cells were pretreated with 5 μg/mL of poly (I:C) for 10 h to trigger IFN induction, followed by SARS-CoV-2 infection for an additional 48 h. Viral replication was significantly reduced after poly (I:C) treatment in Calu-3 cells (Fig. 2D). By contrast, pretreating with poly (I:C) did not affect SARS-CoV-2 replication in ACE2-A549 cells (Fig. 2E). Collectively, the results demonstrate that ACE2 is likely to abort poly (I:C)-mediated inhibition of SARS-CoV-2 replication by antagonizing IFN induction.

10.1128/msphere.00211-22.2 FIG S2 ACE2 antagonizes IFN-I signaling by targeting IRF3 and STAT2. (A) Western blot analysis for exogenous expression of IRF3 in NC- and ACE2-expressing 293T and H1299 cells. (B) RT-qPCR analysis for the expression of IRF3 in NC- and ACE2-expressing A549 cells treated with mock or 5 μg/mL poly (I:C) or SeV (MOI = 1) for 10 h. (C) RT-qPCR analysis for the expression of IRF3 in NC- and ACE2-expressing 293T cells transfected with IRF3 for 24 h. (D) Western blot analysis for endogenous expression and phosphorylation of JNK, P38, and ERK in the WT and ACE2-A549 cells treated with control, or 5 μg/mL poly (I:C) for 6 h and 10 h. (E) Effects of ACE2 on IRF3-induced IFN-β promoter activation. ****, *P < *0.0001. (F) RT-qPCR analysis for the expression of STAT2 in NC- and ACE2-expressing A549 cells treated with mock or 1000 U/mL IFNα for 4 h. ns, no significance; ****, *P < *0.0001. (G) Immunofluorescence image for ACE2 expression and intracellular localization in ACE2-A549 cells treated with or without SeV (MOI = 1) for 6 h; Blue, DAPI; Red, ACE2. Scale bars, 10 μm. Download FIG S2, TIF file, 1.2 MB. Copyright © 2022 Chen et al. 2022 Chen et al. https://creativecommons.org/licenses/by/4.0/ This content is distributed under the terms of the Creative Commons Attribution 4.0 International license.

To further investigate the underlying mechanisms by which ACE2 regulates the IFN response, we stimulated WT A549 and ACE2-A549 cells with IFN-α. WB and RT-qPCR analyses revealed that mRNA level (Fig. S2F), endogenous protein, and phosphorylation level of STAT2 (Fig. 2F) were significantly depressed in the indicated time points, whereas STAT1 phosphorylation was slightly reduced, and IRF9 was not inhibited (Fig. 2F). In addition, the nuclear translocation of STAT2 was completely blocked in ACE-A549 cells (Fig. 2G), indicating that ACE2 was likely to suppress IFN-α-triggered ISGs activation by degrading endogenous STAT2, blocking STAT2 phosphorylation and translocation. Consistent with this observation, exogenous expression of ACE2 aborted IFN-α-mediated inhibition of SARS-CoV-2 (Fig. 2H and I) and Zika virus (Fig. 2J) infection. Of note, dACE2 could also antagonize the antiviral effects of IFNα (Fig. 2J). These data further confirmed that both full-ACE2 and dACE2 could play a critical role in antagonizing IFN-I-mediated antiviral responses.

Interestingly, we noticed that ACE2 was highly expressed in the nucleus after stimulating with poly (I:C) (Fig. 2C), IFNα (Fig. 2G), or SeV (Fig. S2G). Moreover, RNA-Seq analysis revealed that ACE2 expression inhibited the transcription of plentiful host genes after poly (I:C) or IFNα stimulation compared to non-ACE2 expressing NC-cells (Fig. 1J and K). Thus, it is speculated that ACE2 nuclear translocation is likely to participate in transcriptional regulation, and it will be important to address the mechanistic insights into the regulating effects in future studies.

## DISCUSSION

Altogether, we uncovered that human ACE2, which was identified as a primary receptor for SARS-CoV-2 entry, can abort the IFN-I induction and the antiviral effects of the IFN-I response signaling pathway during SARS-CoV-2 infection (Fig. 2K). Moreover, both full-length and truncated ACE2 can suppress the IFN-I signaling. Our data provide mechanistic insight into the distinctive role of ACE2 in promoting SARS-CoV-2 infection in human lung cells and enlightens us that the development of interventional strategies might be further optimized to interrupt ACE2-mediated suppression on IFN-I and its signaling pathway. The detailed molecular basis of the interaction between ACE2 and IFN-related signaling pathways remains to be explored in future studies.

## MATERIALS AND METHODS

### Cell lines and viruses.

A549, HEK293T, NCI-H1299, and Vero E6 cell lines were purchased from the American Type Culture Collection (ATCC, USA). Calu-3 cell line was gifted from Zhengli Shi (Wuhan Institute of Virology, Wuhan, People's Republic of China). The U-251 MG cell line was purchased from BeNa Culture Collection (BNCC). A549, HEK293T, U-251 MG, and Vero E6 cells were cultured in DMEM (Gibco) supplemented with 10% FBS, 100 IU/mL of penicillin, and 100 μg/mL of streptomycin. Calu-3 cells were grown and propagated in Dulbecco's modified Eagle's-nutrient mixture F-12 medium (Gibco) supplemented with 15% fetal bovine serum. NCI-H1299 cells were cultured in RPMI 1640 (Gibco) supplemented with 10% FBS (Gibco), 100 IU/mL of penicillin, and 100 μg/mL of streptomycin. All cells were maintained at 37°C in a fully humidified atmosphere containing 5% CO_2_. SARS-CoV-2/SH01/human/2020/CHN (GenBank accession number: MT121215) was propagated and titrated in Vero E6 cells and used for authentic virus-based infection assay.

### Plasmids and molecular cloning.

Human ACE2 lentiviral cDNA ORF Clone (C-GFPSpark tag, Human, HG10108-ACGLN) mammalian expression plasmids were purchased from Sino Biological. sgRNA against ACE2 (sg-ACE2: ATGAGCACCATCTACAGTAC) were synthesized and cloned into the pLenti-V2 vector. pLV-linker-GFP control vector was cloned from ACE2 lentiviral cDNA ORF Clone as the NC (Fig. 1O). The lentivirus was packaged in HEK293T cells with psPAX2 and pMD2.G, condensed by ultra-centrifugation. Knock-out (KO) cells were selected with puromycin (2 μg/mL) for 14 days, sub-cloned to form single colonies, and validated by Western blotting assay to verify the loss of ACE2 expression. full-ACE2-GFP, dACE2-GFP, or linker-GFP expressed A549/U-251 MG/H1299/HEK-293T cells were sorted by fluorescence-activated cell sorting (FACS) (Fig. S1D).

### Immunofluorescence staining.

Cells on slides were fixed with 4% paraformaldehyde (PFA) for 20 min at room temperature and were permeabilized with 0.1% Triton X-100 in 1 × PBS for 5 min and blocked with blocking buffer (1% BSA and 2% donkey serum diluted in PBS) for 30 min. Immunofluorescence analyses of SARS-CoV-2-infected cells were performed using a rabbit anti-SARS-CoV-2 spike S/S2 protein antibody (1:500, 40590-T62, S&B), a mouse anti-dsRNA antibody (1:200, 10010200, J2-1909, SCICONS), an anti-ACE2 antibody (1:100, A12737, Abclonal), IRF-3 (D6I4C) XP rabbit mAb (1:300, #11904, CST), Alexa Fluor 680 donkey anti-rabbit IgG (H+L) (1:1000, ab175772, Abcam), and Alexa Fluor 555 donkey anti-mouse IgG (H+L) (1:1000, ab150106, Abcam). All cells were mounted with ProLongTM Gold Antifade with DAPI (Life Technologies, P36931) and imaged with a TissueFAXS 200 flow-type tissue cytometer (TissueGnostics GmbH, Vienna, Austria). All statistical analyses of immunofluorescence staining present the results from at least 3000 cells per replicate, and data are shown as the mean ± s.e.m.

### Western blot (WB) analysis.

Cells were lysed using 4 × SDS loading buffer and denatured at 95°C for 10 min. Protein samples were resolved by SDS-PAGE, transferred to PVDF membranes (GE Healthcare), and processed for Western blotting. Western blot (WB) detection of IRF3, P-IRF3, IRF7, P-IRF7, TBK1, P-TBK1, STAT1, P-STAT1, STAT2, P-STAT2, IRF9, and ACE2 was performed using IRF-3 (D6I4C) XP rabbit mAb (1:1000, #11904, CST), Phospho-IRF-3 (Ser396) (D6O1M) rabbit mAb (1:1000, #29047, CST), IRF-7 rabbit mAb (1:1000, #4920, CST), Phospho-IRF-7 (Ser471/472) antibody (1:1000, #5184, CST), TBK1/NAK (E8I3G) rabbit mAb (1:1000, #38066, CST), Phospho-TBK1/NAK (Ser172) (D52C2) XP rabbit mAb (1:1000, #5483, CST), STAT1 (D1K9Y) rabbit mAb (1:1000, #14994, CST), Phospho-STAT1 (Tyr701) (58D6) rabbit mAb (1:1000, #9167, CST), STAT2 (D9J7L) rabbit mAb (1:1000, #72604, CST), Phospho-STAT2 (Tyr690) (D3P2P) rabbit mAb (1:1000, #88410, CST), IRF9 (ISGF-3γ p48) mouse mAb (10 μg/mL, sc-365893, Santa Cruz), and an anti-ACE2 antibody (1:1000, A12737, Abclonal). GAPDH was used as a loading control.

Other antibodies used in the study included: anti-GAPDH (1:3000, AC002, Abclonal), goat anti-rabbit IgG-HRP (1:5000, B2615, Santa Cruz Biotechnology), and HRP Goat Anti-Mouse IgG (H+L) (1:5000, AS003, Abclonal).

### Authentic SARS-CoV-2 infection of human cells.

Authentic SARS-CoV-2 was isolated from a patient in Shanghai with COVID-19. We plaque-purified and massively expanded the initial generation in Vero-E6 with TPCK-treated trypsin at a concentration of 2 μg/mL and stored the virus at −80°C. We deep-sequenced the strain and named it SARS-CoV-2/human/CHN/Shanghai_CH-03/2020 (GenBank accession number: MT622319.1). Compared with the Wuhan strain (GenBank accession number: NC_045512), this strain has the same gene sequence encoding the S glycoprotein. All authentic SARS-CoV-2 infection assays were performed using these early passages of SARS-CoV-2 to ensure the consistency of our experiments. WT or ACE2 expressed A549 and Calu-3 cells were seeded in the 8-well plates (4 × 10^4^ cells per well) in DMEM or DMEM/F12 containing 10% FBS. The cells were infected with 8 × 10^4^ PFU/mL SARS-CoV-2 (MOI = 2) at 37°C for 1 h, which were pre-incubated with control, poly (I:C) (5 μg/mL), or SeV (MOI = 1) at 37°C for 10 h, then washed with 1 × PBS three times and cultured in complete medium for 48 h. All the experiments were performed in BSL-3 labs.

### Viral attachment and entry assay.

For the virus binding assay, 1 × 10^5^ WT and ACE2-A549 cells were seeded in a 12-well plate and cultured for 24 h, and then incubated with SARS-CoV-2 (MOI = 10) in a cold opti-MEM medium on ice for 1 h. Unbound virus was removed and washed three times with cold 1 × PBS. Then, cell lysates were harvested for vRNA quantitation by RT-qPCR. For the virus entry assay, 1 × 10^5^ WT and ACE2-A549 cells were seeded in a 12-well plate and cultured for 24 h, and were then infected with SARS-CoV-2 (MOI = 10) in cold opti-MEM medium on ice for 1 h. Unbound virus was removed and washed three times with cold 1 × PBS. Then, a pre-warmed medium was added to the cells to initiate SARS-CoV-2 internalization for 1 h at 37°C. Cell lysates were harvested for vRNA quantitation by RT-qPCR. All the experiments were performed in BSL-3 labs.

### Poly (I:C) and IFN-α inhibition assay.

For the poly (I:C) inhibition experiment, Calu-3 and ACE2-A549 cells were seeded in 8-well plates (4 × 10^4^ cells per well) and cultured for 24 h in DMEM or DMEM/F12 containing 10% FBS. The cells were then transfected with or without 5 μg/mL poly (I:C) (P9582, Sigma-Aldrich) in DMEM supplemented with 2% FBS for 10 h at 37°C. Cells were then infected with SARS-CoV-2 (MOI = 2) for 2 h at 37°C. The supernatant was removed, and cells were washed three times with 1 × PBS, and then pre-warmed medium containing 2% FBS was added to the cells for another 48 h.

For the IFN-α inhibition experiment, Calu-3 and ACE2 expressed A549 cells were seeded in 8-well plates (4 × 10^4^ cells per well) and cultured 24 h in DMEM or DMEM/F12 containing 10% FBS. Then, cells were pre-incubated with or without 1000 U/mL or 100 U/mL IFN-α (SRP4595, Sigma-Aldrich) in DMEM supplemented with 2% FBS for 10 h at 37°C. Cells were then infected with SARS-CoV-2 (MOI = 2) for 2 h at 37°C. The supernatant was removed, and cells were washed with 1 × PBS three times. Pre-warmed medium containing 2% FBS was then added to the cells for another 48 h. All the slides were prepared for Immunofluorescence analysis. All the experiments were performed in BSL-3 labs.

### Quantitative real-time PCR (RT-qPCR).

According to the manufacturer's instructions, RNA was extracted using an RNEasy RNA isolation kit (Qiagen). One microgram of RNA was transcribed into cDNA using random primers and the Moloney murine leukemia virus reverse transcriptase (Promega, Charbonnieres, France). RT-qPCR was performed using the resulting cDNA templates and GoTaq qPCR Master Mix (Promega) in an Applied Biosystems 7300 real-time PCR cycler (see the Supplementary Information for the primer sequences). The PCR data were analyzed using SDS software (Applied Biosystems). GAPDH expression was used as an internal control. For the SARS-CoV-2 infection experiments, gene expression was normalized to that in unstimulated WT cells unless otherwise noted. All presented RT-qPCR data are the results from more than three biological replicates. All the primers are listed in Table S1.

10.1128/msphere.00211-22.3 TABLE S1 List of primers. Download Table S1, DOCX file, 0.1 MB. Copyright © 2022 Chen et al. 2022 Chen et al. https://creativecommons.org/licenses/by/4.0/ This content is distributed under the terms of the Creative Commons Attribution 4.0 International license.

### Cytokine measurement.

To test cytokine secretion in the supernatant, GFP-A549 and ACE2-A549 (clone#6) were treated with mock, poly(I:C) or SeV for 10 h. Cell-free supernatants were collected and stored at −80°C until analysis. According to the manufacturers ' instructions, the concentrations of inflammatory cytokines in the supernatants were assessed by the cytometric bead array (CBA) (BD Biosciences).

### Whole-transcriptome (RNA-Seq) analysis.

Mock and poly (I:C) or IFNα treated GFP-A549 and ACE2-A549 (clone#6) (three replicates in each group) were harvested at 10 h post-stimulation. Whole-transcriptome sequencing was performed by Biowavelet Biotechnology Inc. (Chongqing, China). Differentially expressed genes (DEGs) were identified using the R Bioconductor package 'edgeR'. Trimmed mean of M-values normalization was applied to account for differences in library size among samples. *P*-values were adjusted for multiple tests using a false discovery rate. Significant DEGs were identified with an FDR ≤ 0.05 and a log_2_ (fold change) ≥1. The top 30 genes are listed in Table S2.

10.1128/msphere.00211-22.4 TABLE S2 List of DEGs. Download Table S2, DOCX file, 0.1 MB. Copyright © 2022 Chen et al. 2022 Chen et al. https://creativecommons.org/licenses/by/4.0/ This content is distributed under the terms of the Creative Commons Attribution 4.0 International license.

### Statistical analyses.

Western blotting and immunofluorescence data were obtained from at least three repeated experiments. At least 3,000 fluorescent cells were imaged and counted with a flow-type tissue cytometer to quantify infected cells. More than three replicates were established per sample. The data were analyzed using Prism 7.0 software (GraphPad, USA) and are presented as the means ± s.e.m. Statistical significance between the two groups was determined by an unpaired two-tailed Student's *t* test. Multiple group comparisons were performed using a two-way analysis of variance (ANOVA). Differences were considered to be significant for *P* < 0.05 (indicated with an asterisk [*]).
