# Supplementary figures and images for: Angiotensin-Converting Enzyme 2 Potentiates SARS-CoV-2 Infection by Antagonizing Type I Interferon Induction and Its Down-Stream Signaling Pathway
Source: mSphere. 2022 Jul 12;7(4):e00211-22. doi: 10.1128/msphere.00211-22 (PMC9429913; doi:10.1128/msphere.00211-22)

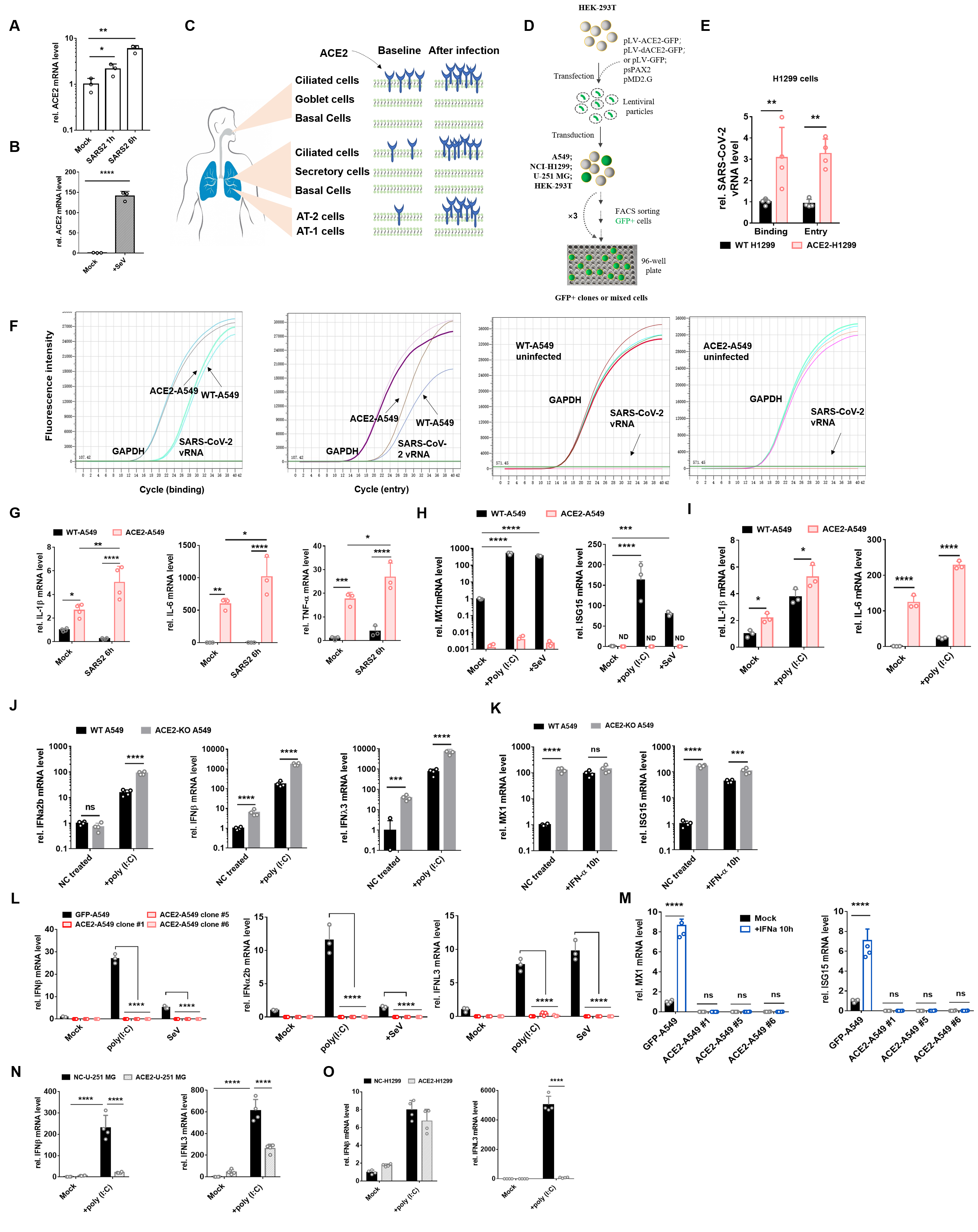

Supplement: FIG S1 [file msphere.00211-22-s0001.tif]

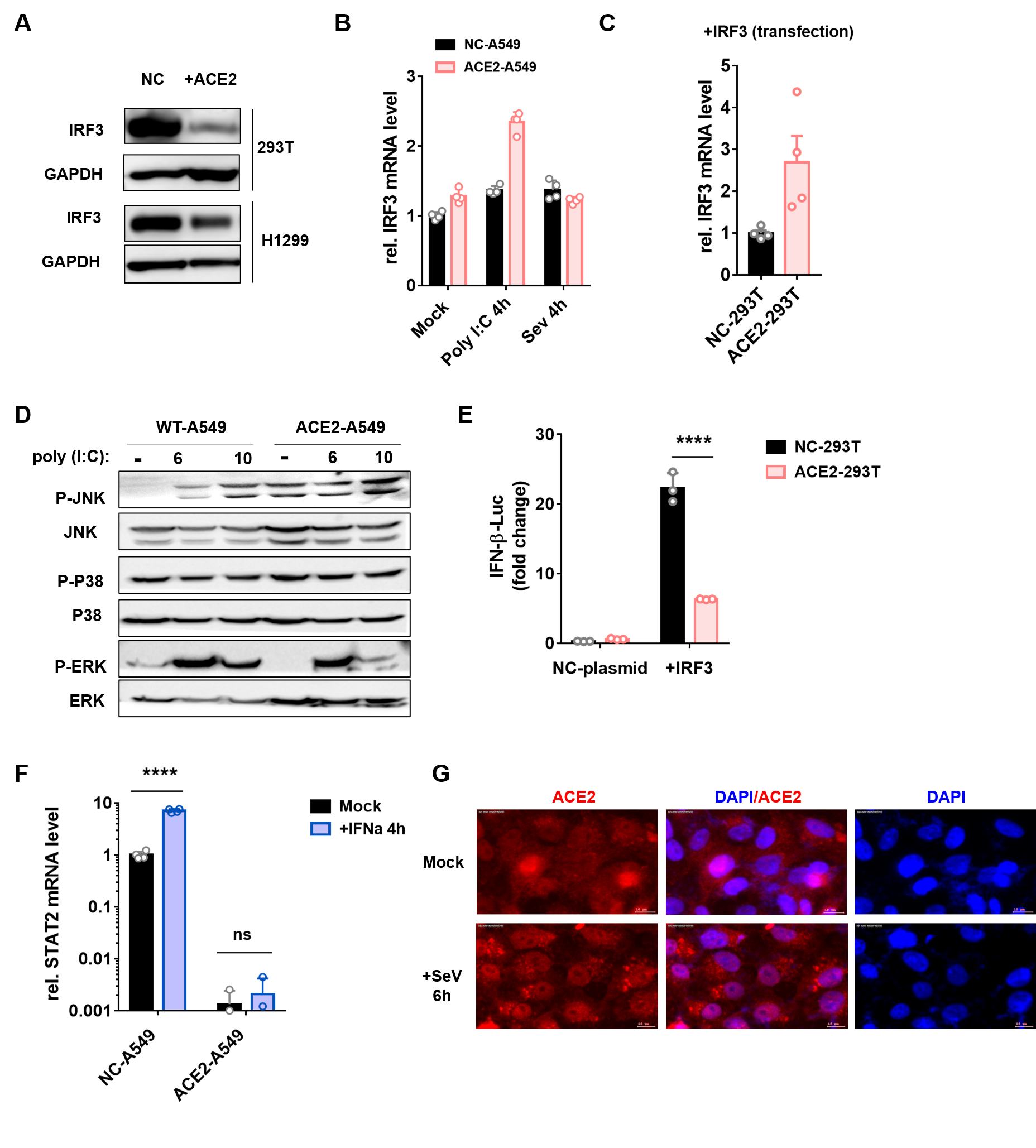

Supplement: FIG S2 [file msphere.00211-22-s0002.tif]
