# Supplementary material for: Angiotensin-Converting Enzyme 2 Potentiates SARS-CoV-2 Infection by Antagonizing Type I Interferon Induction and Its Down-Stream Signaling Pathway
Source: mSphere. 2022 Jul 12;7(4):e00211-22. doi: 10.1128/msphere.00211-22 (PMC9429913; doi:10.1128/msphere.00211-22)
Supplement: TABLE S1 [file msphere.00211-22-s0003.docx]

**Table S1. List of primers**

| **Primer name** | **Primer sequence** |
| --- | --- |
| h-IL6-F | AGACAGCCACTCACCTCTTCAG |
| h-IL6-R | TTCTGCCAGTGCCTCTTTGCTG |
| h-TNFα-F | CTCTTCTGCCTGCTGCACTTTG |
| h-TNFα-R | ATGGGCTACAGGCTTGTCACTC |
| h-ACE2-F | TCCATTGGTCTTCTGTCACCCG |
| h-ACE2-R | AGACCATCCACCTCCACTTCTC |
| h-MX1-F | AGCGGGATCGTGACCAGAT |
| h-MX1-R | TGACCTTGCCTCTCCACTTATC |
| h-ISG15-F | TGGACAAATGCGACGAACCTC |
| h-ISG15-R | TCAGCCGTACCTCGTAGGTG |
| h-IFN-β-F | ATGACCAACAAGTGTCTCCTCC |
| h-IFN-β-R | GGAATCCAAGCAAGTTGTAGCTC |
| h-IFN-α2b-F | GCTTGGGATGAGACCCTCCTA |
| h-IFN-α2b-R | CCCACCCCCTGTATCACAC |
| h-IFN-λ3-F | TAAGAGGGCCAAAGATGCCTT |
| h-IFN-λ3-R | CTGGTCCAAGACATCCCCC |
| h-IL-1β-F | CCACAGACCTTCCAGGAGAATG |
| h-IL-1β-R | GTGCAGTTCAGTGATCGTACAGG |
| SARS-CoV-2-NP-F | ATGCTGCAATCGTGCTACAA |
| SARS-CoV-2-NP-R | GACTGCCGCCTCTGCTC |
| total-ACE2-F | TCCATTGGTCTTCTGTCACCCG |
| total-ACE2-R | AGACCATCCACCTCCACTTCTC |
| full-ACE2-F | GGGCGACTTCAGGATCCTTAT |
| full-ACE2-R | GGATATGCCCCATCTCATGATG |
| dACE2-F | GGAAGCAGGCTGGGACAAA |
| dACE2-R | AGCTGTCAGGAAGTCGTCCATT |
