# Supplementary material for: Angiotensin-Converting Enzyme 2 Potentiates SARS-CoV-2 Infection by Antagonizing Type I Interferon Induction and Its Down-Stream Signaling Pathway
Source: mSphere. 2022 Jul 12;7(4):e00211-22. doi: 10.1128/msphere.00211-22 (PMC9429913; doi:10.1128/msphere.00211-22)
Supplement: TABLE S2 [file msphere.00211-22-s0004.docx]

**Table S2.** **List of DEGs**

**2.1 up-regulated in NC-A549 treated with poly(I:C)**

| **Gene Symbol** | **Entrezid** | **baseMean** | **log2FoldChange** | **pvalue** |
| --- | --- | --- | --- | --- |
| IFNL2 | 282616 | 3213.27 | 11.36 | 2.17E-120 |
| IFNL1 | 282618 | 6088.68 | 11.28 | 5.70E-231 |
| EGR2 | 1959 | 171.03 | 11.15 | 9.79E-21 |
| OR52A1 | 23538 | 168.63 | 11.13 | 1.10E-20 |
| IFNB1 | 3456 | 4112.46 | 11.08 | 7.19E-176 |
| XAF1 | 54739 | 526.53 | 10.92 | 2.02E-26 |
| IFNL3 | 282617 | 3837.91 | 10.53 | 5.53E-32 |
| C2CD4A | 145741 | 109.51 | 10.50 | 2.26E-18 |
| RFPL4A | 342931 | 97.79 | 10.34 | 9.70E-18 |
| ACTN2 | 88 | 94.96 | 10.30 | 1.51E-17 |
| CXCL10 | 3627 | 1071.42 | 10.13 | 4.11E-75 |
| RTP4 | 64108 | 81.40 | 10.08 | 7.42E-17 |
| RUFY4 | 285180 | 81.27 | 10.07 | 1.03E-16 |
| RANBP3L | 202151 | 76.51 | 9.99 | 1.76E-16 |
| APOBEC3G | 60489 | 67.41 | 9.80 | 7.71E-16 |
| RTL9 | 57529 | 67.10 | 9.80 | 7.93E-16 |
| RSAD2 | 91543 | 2236.91 | 9.75 | 4.31E-183 |
| CSAG3 | 389903 | 64.47 | 9.74 | 1.35E-15 |
| FAP | 2191 | 55.38 | 9.53 | 7.94E-15 |
| TULP2 | 7288 | 53.87 | 9.49 | 1.53E-14 |
| CCL4 | 6351 | 580.92 | 9.47 | 3.22E-56 |
| IDO1 | 3620 | 2440.59 | 9.32 | 1.23E-232 |
| EGR4 | 1961 | 168.52 | 9.27 | 6.23E-19 |
| NXF3 | 56000 | 44.43 | 9.20 | 9.07E-14 |
| CXCL11 | 6373 | 1088.30 | 9.14 | 4.76E-16 |
| DHX58 | 79132 | 687.98 | 9.13 | 6.35E-77 |
| HIST1H2BG | 8339 | 40.94 | 9.09 | 2.28E-13 |
| IFIT2 | 3433 | 40691.27 | 9.08 | 0 |
| CH25H | 9023 | 439.21 | 9.07 | 2.88E-51 |
| RIPOR2 | 9750 | 39.57 | 9.04 | 3.87E-13 |

**2.2 up-regulated in NC-A549 treated with IFNα**

| **Gene Symbol** | **Entrezid** | **baseMean** | **log2FoldChange** | **pvalue** |
| --- | --- | --- | --- | --- |
| XAF1 | 54739 | 383.4001655 | 10.13645564 | 6.18E-23 |
| RUFY4 | 285180 | 31.89384216 | 8.402835718 | 1.42E-11 |
| IFITM1 | 8519 | 669.8225671 | 8.23554479 | 2.81E-88 |
| OAS2 | 4939 | 3992.468603 | 7.958878206 | 0 |
| RTP4 | 64108 | 20.40111988 | 7.758184567 | 1.54E-09 |
| SLC15A3 | 51296 | 113.3172343 | 7.365672766 | 2.63E-22 |
| IFI27 | 3429 | 3364.491284 | 7.138712866 | 9.51E-116 |
| BST2 | 684 | 706.5363101 | 6.925913883 | 7.47E-145 |
| MX1 | 4599 | 4264.350778 | 6.897980526 | 0 |
| IFI44L | 10964 | 637.1320826 | 6.765760126 | 3.76E-138 |
| CETP | 1071 | 9.728773592 | 6.689607884 | 1.27E-06 |
| ISG15 | 9636 | 8469.064758 | 6.612235745 | 0 |
| RSAD2 | 91543 | 318.2101516 | 6.602960325 | 1.45E-72 |
| IFIT1 | 3434 | 13718.9109 | 6.598687975 | 0 |
| MX2 | 4600 | 593.73634 | 6.42501015 | 5.56E-140 |
| APOBEC3G | 60489 | 7.461346477 | 6.306756606 | 1.36E-05 |
| CARD16 | 114769 | 7.15922916 | 6.246598898 | 1.97E-05 |
| CD74 | 972 | 25.42144944 | 6.22102411 | 3.48E-08 |
| ACTN2 | 88 | 6.763776184 | 6.166309274 | 3.59E-05 |
| LAMP3 | 27074 | 922.7712559 | 6.155398872 | 4.60E-231 |
| IFI6 | 2537 | 6303.33768 | 6.143977883 | 7.17E-251 |
| CMPK2 | 129607 | 1535.406459 | 6.032515637 | 0 |
| OASL | 8638 | 2201.950134 | 6.022017523 | 0 |
| IFIT3 | 3437 | 7217.666245 | 5.852786784 | 2.45E-137 |
| CXCL10 | 3627 | 67.87828263 | 5.79080762 | 3.92E-21 |
| SERPING1 | 710 | 92.18353624 | 5.748076644 | 1.87E-27 |
| DHX58 | 79132 | 81.55745894 | 5.71005762 | 3.23E-25 |
| IDO1 | 3620 | 252.4518283 | 5.695103401 | 9.52E-73 |
| ANGPTL1 | 9068 | 4.569115146 | 5.598243509 | 0.000509938 |
| IFI44 | 10561 | 1252.861362 | 5.424136814 | 0 |

**2.3 up-regulated in ACE2-A549 treated with poly(I:C)**

| **Gene Symbol** | **Entrezid** | **baseMean** | **log2FoldChange** | **pvalue** |
| --- | --- | --- | --- | --- |
| MID2 | 11043 | 133.4920538 | 0.705013864 | 0.002370227 |
| OSGIN1 | 29948 | 157.863475 | 0.685479203 | 0.000802474 |
| MSTO1 | 55154 | 189.5469879 | 0.664231945 | 0.000327461 |
| IFIT3 | 3437 | 399.7352573 | 0.654572161 | 5.19E-07 |
| SGK1 | 6446 | 148.3846911 | 0.618601002 | 0.001931206 |
| HMOX1 | 3162 | 601.4262949 | 0.617261776 | 1.50E-08 |
| ARRDC3 | 57561 | 1526.630842 | 0.616753519 | 3.09E-11 |
| TIMM8B | 26521 | 401.1540254 | 0.602211916 | 5.91E-07 |
| THOC3 | 84321 | 480.4329853 | 0.594880252 | 0.000145455 |
| NDUFB1 | 4707 | 256.5191755 | 0.582528731 | 0.000138227 |
| AKR1B15 | 441282 | 686.5392506 | 0.576061929 | 3.94E-08 |
| INO80C | 125476 | 138.3998892 | 0.5489708 | 0.004203643 |
| DDX60 | 55601 | 195.3893054 | 0.545845101 | 0.000982083 |
| MAD2L1BP | 9587 | 154.9562734 | 0.541893371 | 0.009618426 |
| DSCC1 | 79075 | 309.7545102 | 0.535668588 | 0.000306806 |
| CENPW | 387103 | 401.3465833 | 0.533994949 | 4.01E-05 |
| ARHGEF6 | 9459 | 180.7229716 | 0.527023897 | 0.010580844 |
| ATP5MD | 84833 | 588.0677853 | 0.52362429 | 5.40E-06 |
| RAD18 | 56852 | 219.153058 | 0.520296791 | 0.000936447 |
| FRK | 2444 | 187.1879079 | 0.514516102 | 0.002221785 |
| SRPRB | 58477 | 494.8690674 | 0.512479832 | 0.000183644 |
| CRELD2 | 79174 | 377.1365783 | 0.512305146 | 7.26E-05 |
| SNRPD1 | 6632 | 969.9935209 | 0.510821781 | 1.25E-08 |
| GMPPB | 29925 | 439.7902908 | 0.509774736 | 0.000111209 |
| AKR1B10 | 57016 | 9408.913698 | 0.509055562 | 1.01E-15 |
| CTU1 | 90353 | 138.2353216 | 0.508213679 | 0.034151983 |
| ACSS3 | 79611 | 141.3858709 | 0.497087136 | 0.022367282 |
| BORA | 79866 | 352.9334872 | 0.497063448 | 7.62E-05 |
| LSM8 | 51691 | 296.6771001 | 0.494538745 | 0.002794629 |
| COG6 | 57511 | 329.5950155 | 0.485719242 | 0.000928443 |

**2.2 up-regulated in ACE2-A549 treated with IFNα**

| **Gene Symbol** | **Entrezid** | **baseMean** | **log2FoldChange** | **pvalue** |
| --- | --- | --- | --- | --- |
| FCRLB | 127943 | 3.890265605 | 5.489910437 | 0.001019455 |
| GBP1 | 2633 | 542.7065913 | 5.120302676 | 1.54E-177 |
| GBP6 | 163351 | 2.878559864 | 5.050312431 | 0.003186399 |
| LRRTM2 | 26045 | 9.852908441 | 4.958690712 | 8.33E-05 |
| CXCL10 | 3627 | 82.94292943 | 4.402562125 | 4.89E-31 |
| ABHD1 | 84696 | 3.150420778 | 4.128696413 | 0.014312375 |
| CCDC81 | 60494 | 3.049685061 | 4.087080172 | 0.01486659 |
| RNF212 | 285498 | 2.915586433 | 4.009135589 | 0.029847505 |
| TMEM95 | 339168 | 2.533762432 | 3.835305385 | 0.029699553 |
| MPZ | 4359 | 4.033652573 | 3.621245777 | 0.016079168 |
| BOLA2B | 654483 | 71.94617174 | 3.568719042 | 8.32E-24 |
| C15orf62 | 643338 | 3.73085441 | 3.479931259 | 0.021095795 |
| COLQ | 8292 | 3.484823703 | 3.386162948 | 0.033948508 |
| GBP3 | 2635 | 79.05725615 | 3.32072034 | 4.08E-24 |
| SLC15A3 | 51296 | 5.280246408 | 3.296513968 | 0.00844596 |
| C3 | 718 | 2236.051291 | 3.189101567 | 0 |
| NLRC5 | 84166 | 29.65751165 | 3.124790684 | 1.13E-09 |
| PSMB8 | 5696 | 541.5609294 | 2.958225787 | 7.35E-121 |
| IFI44L | 10964 | 31.87988606 | 2.92973591 | 2.39E-09 |
| BATF2 | 116071 | 59.51902086 | 2.848377752 | 2.14E-16 |
| SAMD9L | 219285 | 111.0723843 | 2.841472001 | 2.33E-25 |
| SMPD3 | 55512 | 3.65572817 | 2.83100063 | 0.037666321 |
| IL18BP | 10068 | 124.6311622 | 2.786482237 | 7.10E-30 |
| GBP2 | 2634 | 849.0809665 | 2.769072746 | 9.19E-158 |
| POU3F1 | 5453 | 7.330480264 | 2.721047453 | 0.008320889 |
| IRF1 | 3659 | 953.1439091 | 2.658098182 | 9.81E-162 |
| CD274 | 29126 | 24.51862503 | 2.558001727 | 1.92E-05 |
| TAP1 | 6890 | 951.0256023 | 2.549625615 | 4.22E-124 |
| CXCL11 | 6373 | 12.62534736 | 2.54539343 | 0.000332833 |
| S100A8 | 6279 | 8.424309144 | 2.530802484 | 0.008932573 |
